# Can AI Reliably Identify Marine Microplastics in Wildlife? Assessing Multi-Modal Foundation Models for Polymer Classification with Minimal Training

**DOI:** 10.3390/ijerph23070929

**Published:** 2026-07-20

**Authors:** Gabriela Fernandez, Domenico Vito, Siddharth Suresh-Babu, Dipsy Booth, Sayali Sanjay Shelke, Kawther Kaziz, Robert Yabumoto, Mohamed Banni

**Affiliations:** 1 Metabolism of Cities Living Lab, Center for Human Dynamics in the Mobile Age, San Diego State University, 5500 Campanile Drive, San Diego, CA 92185, USA; dvito@sdsu.edu (D.V.); siddharthsb06@gmail.com (S.S.-B.);; 2 Laboratory of Agrobiodiversity and Ecotoxicology, Higher Institute of Biotechnology Monastir, University of Monastir, Avenue Taher Hadded, Monastir 5000, Tunisia; kawtherkaziz1@gmail.com (K.K.);

**Keywords:** microplastic detection, image segmentation, artificial intelligence, polygon annotation, environmental risk assessment, One Health, mediterranean coastal waters

## Abstract

**Highlights:**

**Public health relevance—How does this work relate to a public health issue?**

- Marine microplastic pollution in the Mediterranean may expose coastal populations through seafood consumption and associated polymer toxicity, while current monitoring methods lack sufficient spatial and temporal coverage for population-level assessment.
- Tunisia’s coastal zone, shaped by urbanization, tourism, and limited water exchange, provides a relevant case study for understanding links between pollution sources and potential human exposure pathways.

**Public health significance—Why is this work of significance to public health?**

- CLIP-based models achieved 93.1% accuracy in classifying microplastic polymers from field images without spectroscopic or expert input at inference time.
- The study supports scalable, polymer-resolved monitoring within a One Health framework linking environmental contamination and potential exposure pathways.

**Public health implications—What are the key implications or messages for practitioners, policy makers and/or researchers in public health?**

- AI-based microplastic classification can complement traditional methods by enabling large-scale, cost-effective coastal surveillance for environmental health monitoring.
- Integrating polymer-resolved AI outputs into One Health systems may support evidence-based coastal management and regional policy coordination in the Mediterranean.

**Abstract:**

While existing AI-based microplastic monitoring studies predominantly rely on task-specific fine-tuned models, this study evaluates whether general-purpose multimodal foundation models with no spectroscopic instrumentation, minimal fine-tuning, and minimal computational resources can serve as accessible, low-cost tools for polymer classification under ecologically realistic field conditions with samples of plastic debris collected from coastal Sousse, Tunisia, a Mediterranean region experiencing anthropogenic pollution pressures. A curated dataset of 1080 high-resolution images was developed, representing six polymer groups (HDPE, LDPE, PA, PET, PP, PS, and mixed plastics). Fragments were imaged under standardized lighting conditions against natural sand backgrounds to preserve environmental realism. Each image was manually annotated using polygon-based boundaries to generate pixel-level segmentation masks and associated class labels, providing expert-validated ground truth for quantitative evaluation. Multimodal LLMs were evaluated using a composite scoring framework. Spatial accuracy was assessed using mean Intersection-over-Union (mIoU) against expert annotations, while polymer classification performance was measured using macro-averaged F1 scores across all categories. Model reliability was further evaluated through prompt stability testing and robustness analyses under controlled environmental perturbations designed to assess consistency across varying coastal imaging conditions. Results indicate that multimodal foundation models can distinguish plastic fragments from sand backgrounds, although performance varied across polymer classes and environmental perturbations. The weighted composite framework provides a structured approach for comparing model performance according to ecological monitoring objectives rather than computational metrics. These findings contribute to understanding the potential utility and current limitations of AI-based approaches for marine microplastic analysis and provide insights into coastal pollution patterns and ecosystem health within a One Health monitoring context.

## 1. Introduction

Microplastic pollution has become a pervasive global challenge impacting marine ecosystems, food systems, and human health. Semi-enclosed seas are particularly vulnerable due to dense coastal populations, tourism, shipping activity, and limited water circulation. Given these conditions, the Mediterranean Sea represents a major hotspot of plastic accumulation and microplastic generation, where historical anthropogenic pressures due to long term intensified contamination and debris degradation contribute to a rise in the concentration of such types of pollution [1]. Situated on the North African side of the enclosed sea, Tunisia’s coastal zones provide a critical case study for understanding how regional pollution sources translate into shoreline microplastic presence at the intersection of land, sea, and communities. These coastal zones are characterized by a combination of urban coastal development, intensive seasonal tourism, fishing activities, and maritime traffic, which together reflect the diversity of anthropogenic pressures observed across the Mediterranean basin.

The increasing concentration of microplastics in the Mediterranean Sea represents a significant One Health risk that threatens biodiversity and ecosystem resilience through processes such as ingestion, chemical adsorption, biofouling, and habitat disruption. These impacts may also affect fisheries, food security, and human health through trophic transfer [2]. Addressing these interconnected impacts requires an integrated One Health approach, which recognizes the interdependence of environmental, animal, and human health and promotes coordinated monitoring, risk assessment, and management strategies across these domains [3].

Such an approach can help identify shared vulnerabilities and support more comprehensive responses to environmental contaminants, including microplastics. Monitoring microplastics, therefore, is essential for integrated environmental management and coastal protection.

Within this context, AI-driven monitoring can serve as an enabling technology for One Health by increasing the speed, consistency, and spatial coverage of environmental surveillance efforts. Early detection and characterization of microplastic contamination in coastal environments can provide timely information on pollution hotspots, support assessment of potential exposure pathways affecting marine organisms, and inform subsequent ecological and public health investigations. Although AI-based image analysis does not directly measure toxicology, biological uptake, or health outcomes, it can facilitate the generation of environmental evidence that supports integrated risk assessment across environmental, animal, and human health domains.

While traditional monitoring methods rely on manual sorting and spectroscopic validation, which are accurate but costly, time-intensive, and difficult to scale [4], advances in artificial intelligence, particularly multimodal and vision-language models, offer new opportunities to automate detection and classification of plastic debris. However, many studies rely on controlled datasets with limited real-world applicability [5]. This study addresses this gap by developing an expert-annotated dataset from Tunisia and evaluating AI models under realistic conditions, supporting scalable monitoring and harmonized Mediterranean strategies [3,6].

## 2. Literature Review

### 2.1. Marine Microplastic Detection Techniques

The detection of marine microplastics is essential for evaluating contamination in aquatic ecosystems, as accurate identification precedes reliable quantification [7]. Traditional methods usually rely on visual sorting under stereomicroscopes, where particles are classified by size, color, and morphology. Although cost-effective and accessible, this approach is subjective and prone to misclassification, especially for particles smaller than 500 μm and fibrous materials resembling organic matter [4,7]. To improve accuracy, pre-treatment techniques such as density separation, sieving, and chemical digestion are used to isolate plastics and reduce interference [8,9,10].

However, these methods cannot confirm polymer composition. Spectroscopic techniques, including FTIR and Raman spectroscopy, provide reliable identification through polymer-specific signatures [4,7]. Despite their precision, they are time-consuming, costly, and difficult to scale for large monitoring efforts [11]. As sample volumes increase, these limitations highlight the need for faster, more reproducible detection methods. Emerging image-based and computational approaches aim to enhance efficiency, reduce bias, and improve consistency, marking a shift toward data-driven environmental monitoring [8,9].

### 2.2. AI in Environmental Monitoring

Artificial intelligence (AI) has become a critical tool in environmental monitoring, enabling the analysis of large and complex datasets across scales [12,13]. Deep learning models, particularly convolutional neural networks (CNNs), have improved image classification and feature extraction in applications such as pollution detection and ecosystem mapping [10]. These approaches enhance scalability, reduce human bias, and increase reproducibility. However, AI systems face challenges, including the need for large, annotated datasets, limited interpretability, and reduced performance under variable environmental conditions [12,13]. While CNNs learn hierarchical visual features directly from image pixels, their performance often depends on large task specific training datasets and may degrade when objects appear under environmental conditions that differ from those represented during training. In the context of marine plastic detection, factors such as weathering, discoloration, partial burial, biofouling, and heterogeneous backgrounds can obscure purely visual cues and complicate polymer identification. To address this, recent research focuses on more adaptable and transferable models, such as multimodal foundation models that integrate visual and semantic data [14]. By combining image representations with semantic information learned from large scale image text datasets, multimodal models can associate visual patterns with higher level concepts and contextual relationships. This capability may improve generalization across diverse environmental conditions and reduce reliance on narrowly defined visual features alone. Consequently, multimodal foundation models offer a promising framework for environmental monitoring tasks where object appearance varies substantially across locations and ecological settings.

### 2.3. Limitations of Traditional Microplastic Monitoring and AI-Based Approaches

Conventional microplastic monitoring methods based on manual sorting and spectroscopic validation, while highly accurate, remain time-consuming, costly, and difficult to scale for large coastal surveys [4]. In recent years, artificial intelligence-based approaches, including deep learning and vision-language models, have emerged as promising tools for automated detection and classification of marine plastic debris. However, most existing approaches rely on small, laboratory-based, or highly controlled datasets, which limit their robustness and generalization to heterogeneous real-world coastal environments [15]. In the context of marine debris detection, controlled datasets typically consist of clean, isolated plastic fragments photographed under standardized laboratory conditions, often using uniform backgrounds, consistent illumination, minimal occlusion, and limited variations in fragment weathering or degradation. In contrast, environmentally realistic datasets incorporate natural substrates, variable lighting conditions, heterogeneous fragment morphologies, weathered surfaces, partial burial, and other visual complexities commonly encountered in field settings. In contrast, multimodal learning frameworks offer the potential to integrate visual and contextual information, improving segmentation and classification performance under variable environmental conditions. In this study, we address these limitations by developing an expert-annotated dataset of coastal plastic fragments collected from Tunisia and evaluating multimodal models under ecologically realistic conditions that preserve natural sand backgrounds, environmental weathering characteristics, and variability in fragment appearance while maintaining consistent image acquisition procedures for qualitative evaluation.

## 3. Materials, Methods, and Results

The study utilized a dataset of 1080 high-resolution images collected along the Mediterranean coast of Sousse, Tunisia, containing various marine debris including microplastics categorized as HDPE, LDPE, PA, PET, PP, PS, mixed, and others. In accordance with the widely accepted definition, microplastics were defined as plastic particles ranging from 1 to 5 mm in size [7]. The images were taken in March, April, and May. To ensure consistency and reduce variability due to environmental imaging conditions, all images were acquired under controlled lighting conditions using a Samsung mobile SM-N975U1 under dry sediment conditions to ensure particle stability and to avoid distortion or reflectance variability associated with moisture. This workflow combines field-derived (environmentally sourced) samples with standardized laboratory imaging conditions to enable robust and reproducible dataset construction for model training.

All microplastic particles were within the 1–5 mm size range and were considered unweathered, as they did not exhibit visible signs of environmental degradation such as surface cracking, discoloration, or biofouling. No systematic quantification of particle size distribution within this range, morphology percentages, or color distribution was performed, as the primary objective of the study was to develop and evaluate a multi-modal model for segmentation and polymer classification rather than to provide a detailed environmental characterization of microplastic pollution. Nevertheless, the dataset includes substantial variability in particle shapes, colors, and polymer types to improve model generalization under ecologically realistic conditions.

Microplastic particles were collected from the shoreline of Chott Mariem beach using an opportunistic manual sampling strategy. No fixed transects or predefined GPS points were used; instead, sampling was conducted along accessible intertidal zones with the objective of capturing the widest possible diversity of plastic debris types and polymers present on the beach. The shoreline was not pre-cleaned prior to sampling. Visible plastic debris was manually collected from surface deposits in the intertidal zone. This approach was selected to maximize variability in polymer types, shapes, and colors for the construction of a robust image dataset suitable for machine learning applications.

### 3.1. Dataset Development

To develop a reliable dataset for model training and evaluation, Roboflow, an image annotation platform, has been used to manually delineate the various microplastic fragments using polygon-based segmentation. Pixel tracing was selected instead of bounding boxes to better capture the irregular shapes and fine contours characteristic of environmental debris. As particle appearance varied across polymer types, annotation procedures were adapted accordingly. Thin film fragments, particularly those associated with low density plastics, were the most difficult to segment due to their translucent surfaces, folds, and weak contrast against sand backgrounds. These masks often required multiple refinements. In contrast, more rigid polymers such as PET and PS typically displayed clearer edges, allowing more direct boundary tracing, although surface reflections occasionally produced misleading contours that needed correction. Finally, all annotations were reviewed at high magnification and iteratively corrected, when necessary, especially in cases involving clustered or partially overlapping particles (i.e., sand). The validated masks formed the ground benchmark used to evaluate automated segmentation outputs, with intersection over union serving as the primary accuracy metric for comparing model predictions against expert defined boundaries [16,17,18]. No chemical validation using Fourier-transform infrared spectroscopy (FTIR) or Raman spectroscopy was performed prior to annotation; instead, polymer classification was based on visual inspection and morphological characteristics following established field-based identification practices commonly used in image-based microplastic studies, particularly in the context of machine learning dataset development. Polymer assignment was performed during field collection based on macroscopic characteristics and the presumed origin of common plastic items (e.g., bottles, bags, packaging materials), following established field-based identification practices in microplastic research. This approach allowed preliminary classification into polymer categories (e.g., PET, LDPE, PP) based on typical usage and visual characteristics observed at the time of collection [19]. However, it is acknowledged that visual and source-based identification does not provide chemical confirmation of polymer composition, and this limitation is addressed in the discussion.

### 3.2. Evaluation Subset, Shared Protocol and Model Overview

From the full 1080-image dataset, a balanced evaluation subset of 60 images was constructed, comprising ten images per polymer class across six categories: HDPE, LDPE, PA, PET, PP, and PS. The mixed plastics category was excluded to preserve well-defined classification boundaries. Images were drawn from each class using a fixed random seed (42) to ensure reproducibility across all model runs. All models were evaluated on RGB pixel data only, with no spectroscopic, chemical, or multispectral input features, reflecting the study’s core constraint of visually driven monitoring under ecologically realistic field conditions. Classification performance was assessed using macro-averaged F1 scores across all six classes, with spatial segmentation accuracy measured via mean Intersection-over-Union (mIoU) against expert-validated polygon masks.

Four multimodal foundation models were evaluated: LLaVA-OneVision, Qwen2-VL, Llama-3.2-Vision-Instruct, and CLIP ViT-B/32. The first three are autoregressive vision-language models that generate natural language responses conditioned on image and text inputs; CLIP operates through contrastive visual similarity matching against precomputed class prototypes and requires no textual prompt.

### 3.3. Phase 1: Generative Vision-Language Models

The first phase of the evaluation tested whether large pretrained vision-language models, without any task-specific fine-tuning, could identify polymer types directly from field images through natural language reasoning. Three models were selected to represent a range of architectures and parameter scales: LLaVA-OneVision (7 billion parameters), Qwen2-VL (2 billion parameters), and Llama-3.2-Vision-Instruct (11 billion parameters). A key hypothesis underlying this phase was that these models, having been trained on vast multimodal corpora, might possess sufficient embedded visual and materials knowledge to perform zero-shot polymer classification when guided by a well-constructed prompt. This hypothesis motivated significant effort in prompt design, as the output quality of all three models proved highly sensitive to how the classification task was framed.

#### 3.3.1. LLaVA-OneVision

LLaVA-OneVision (llava-onevision-qwen2-7b-ov-hf) couples a visual encoder with a Qwen2-7B language backbone and was loaded via the Hugging Face image-text-to-text pipeline with float16 precision and automatic GPU device mapping. Images were passed at native field resolution without resizing. Inference was capped at 256 new tokens per image, with outputs parsed through a two-stage JSON extraction procedure. Results were saved in both CSV and JSON formats.

LLaVA produced the most distributed misclassification pattern of the three generative models, but its overall performance remained poor. Analysis revealed that 44 of the 60 predictions were misclassified and a deeper analysis revealed two dominant confusion pairs: PET was redirected to LDPE in 18 cases, and PP was split between LDPE (11 cases) and HDPE (9 cases). The remaining six errors comprised PP misclassified as PET (2 cases), LDPE misclassified as HDPE (2 cases), and PS misclassified as LDPE (2 cases), accounting for all 44 errors in full. Taken together, these results reflect a consistent pattern of convergence toward HDPE and LDPE, suggesting that the model anchors on coarse surface-level visual heuristics that map most polymer appearances onto these two visually dominant classes. In all 44 errors, the model returned a confidence score of 1.0, demonstrating a complete failure of uncertainty calibration. The model expressed maximum certainty on every incorrect prediction, rendering its confidence output uninformative for any downstream filtering or quality control workflow.

#### 3.3.2. Qwen2-VL

Qwen2-VL-2B-Instruct was loaded with 4-bit NF4 quantization with double quantization enabled and float16 compute, reducing the model footprint approximately eightfold relative to full precision. Images were processed in batches of 8 with GPU memory cleared between batches.

Despite receiving the most descriptive and carefully engineered prompt of any model in this study, Qwen2-VL produced the worst classification results overall. The model collapsed nearly all predictions onto a single class, HDPE, regardless of the true polymer present, achieving a recall of 1.00 for HDPE and 0.00 for all other classes, with an overall accuracy of 28.6% and a macro F1 of 0.07. This outcome illustrates a fundamental limitation of prompt engineering as a substitute for model capacity: the 2 billion parameter scale was insufficient to leverage the embedded visual decision framework effectively, and the model defaulted to the class whose broad visual description, rigid, opaque, thick, most closely matched its general representation of a plastic fragment on sand.

#### 3.3.3. Llama-3.2-Vision-Instruct

Llama-3.2-11B-Vision-Instruct (meta-llama/Llama-3.2-11B-Vision-Instruct) is the largest generative model evaluated and uses a cross-attention architecture in which visual features are injected into the language model via dedicated cross-attention layers, rather than being interleaved as visual tokens. The model was loaded with 4-bit NF4 quantization on GPU and ran on a local Windows workstation, reflecting the larger memory demands of the 11 B parameter model. Images were down sampled to a maximum of 512 × 512 pixels prior to inference, the only preprocessing reduction applied across any model in this evaluation.

The prompt followed the same four-field JSON schema as LLaVA but extended the label set to include mixed and unknown, allowing the model to express classification ambiguity rather than forcing a committed prediction. Results were written to both an aggregate JSON file and individual per-image JSON files. An analysis of misclassification errors produced by the Llama 3.2 reveals two distinct failure patterns: a strong systematic bias toward predicting HDPE, and a smaller set of cases in which the model returned no classification at all. Every plastic category in the evaluation set was misclassified as HDPE. PET and PS each produced 10 HDPE predictions, followed by OTHER (9), PP (9), and LDPE (8). Notably, some of these substitutions involve plastics that are visually dissimilar to HDPE under normal imaging conditions. A less frequent error pattern involved the model returning ‘unknown’ rather than committing to a plastic type. This occurred five times in total. The systematic HDPE bias reduces the model’s practical utility across all plastic categories and is unlikely to be resolved through prompt engineering alone.

### 3.4. Phase 2: CLIP ViT-B/32—Visual Similarity Matching

The poor performance of the generative models motivated a fundamentally different approach to the classification problem. Rather than asking a model to reason about polymer identity through language, the second phase evaluated whether a model trained to learn a shared visual embedding space, without any language generation at inference time, could match field images to reference polymer classes more reliably. CLIP ViT-B/32 (OpenAI weights, accessed via open-clip-torch) was selected for this purpose.

#### 3.4.1. Why CLIP Represents a Different Paradigm

CLIP was pretrained contrastively on approximately 400 million image-text pairs, learning to place visually similar images close together in a 512-dimensional embedding space regardless of superficial variation in lighting, orientation, or background. Critically, this pretraining produces a visual feature extractor that encodes texture, shape, and material properties at a level of abstraction that generalizes across imaging conditions, without requiring a prompt, without generating text, and without being sensitive to how a task is verbally framed. This eliminates the prompt engineering bottleneck that dominated the generative model phase and replaces it with a single design decision: the construction of representative per-class prototype embeddings from reference images.

#### 3.4.2. Reference Prototype Construction

For each of the six polymer classes, ten images were randomly selected using a fixed random seed (42) and passed through CLIP’s visual encoder to produce 512-dimensional L2-normalized embedding vectors. The ten per-class vectors were averaged element-wise and the mean re-normalized, yielding a single prototype representing the average visual appearance of each polymer class in CLIP’s embedding space. Images were preprocessed using CLIP’s built-in pipeline, resized to 224 × 224 pixels and normalized before encoding. All computations were performed with gradient calculation disabled on a T4 GPU.

#### 3.4.3. Classification and Ground Truth

**Figure 1:**
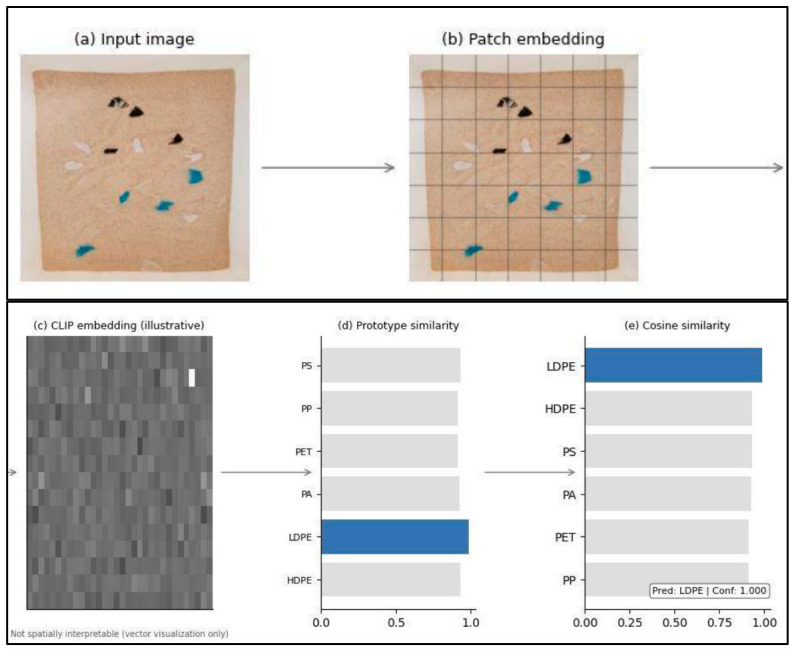
CLIP model process breakdown (illustrative). The 5 panels are described: (**a**) Input image (true label LDPE): shows the original uploaded image resized to 224 × 224. (**b**) Patch embedding: same image with a grid overlay, illustrating how CLIP’s Vision Transformer slices the image into patches before processing. (**c**) CLIP embedding: a heatmap-style visualization of the raw 512-dimensional embedding vector reshaped into a 16 × 32 grid (purely illustrative, not spatially meaningful). (**d**) Prototype similarity: horizontal bar chart comparing the image embedding’s cosine similarity against pre-computed “prototype” embeddings for each target class. (**e**) Cosine similarity: similar bar chart but sorted, with the predicted class highlighted in blue and a confidence annotation (LDPE).

At inference time, each test image was embedded using the same pipeline and its cosine similarity to all six class prototypes was computed. The class with the highest similarity score was assigned as the prediction. Raw cosine scores were min-max normalized to [0,1] across classes to produce a per-prediction confidence value. Figure 1 illustrates how the CLIP model predicts image labels.

**Figure 2:**
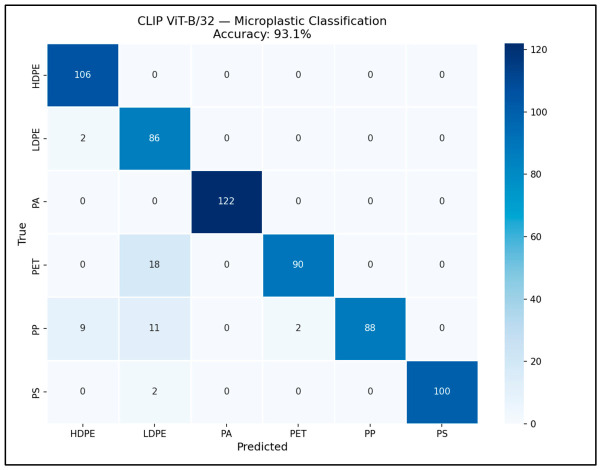
CLIP model results (confusion matrix).

Unlike the generative models, which were evaluated on the 60-image balanced subset, CLIP was applied across 636 test samples from the annotated dataset derived from YOLO-format annotation files. For images containing multiple fragments, the dominant class, defined as the most frequently occurring non-other annotation, was used as the ground truth label. Performance was assessed with a full confusion matrix (Figure 2 below shows the confusion matrix), as well as per-class precision, recall, and F1 scores. The model achieved 93.1% overall accuracy under the specific imaging and annotation conditions represented in the dataset. However, because both the prototype representations and test images originated from the same dataset and imaging environment, this performance should be interpreted as an estimate of within-dataset classification accuracy and may not fully reflect generalizability to independent datasets or different imaging conditions. PA, HDPE, and PS were identified almost flawlessly; the model essentially never confuses these with other plastics. PP and PET were the weakest, with 80–83% accuracy. The model frequently mistook them for LDPE. LDPE acted as a “catch-all”; it correctly caught nearly all real LDPE samples, but also absorbed misclassified PP and PET samples. For more information, see the Supplementary Material.

Table 1, below, provides a quick reference to the architecture and the functioning of each of the models considered.

## 4. Discussion

### 4.1. Prompt Engineering as a Central Methodological Challenge

A consistent and practically significant finding across all three generative models was that classification performance depended critically on prompt construction. Initial prompts that simply asked the model to identify the polymer type from a list of labels produced unstable and largely inaccurate outputs. Models either failed to commit to a label, returned responses outside the permitted label set, or produced valid JSON with incorrect classifications that showed no systematic relationship to the true polymer class. This necessitated iterative prompt refinement across multiple experimental cycles.

For LLaVA-OneVision and Llama-3.2-Vision-Instruct, the final prompts converged on a four-field JSON schema (has_plastic, predicted_label, confidence, and reason) accompanied by explicit output formatting rules prohibiting markdown, role labels, and any text outside the JSON object. For Qwen2-VL, the prompt was substantially more elaborate, embedding a full visual decision guide that described the physically diagnostic properties of each polymer class, including thickness, rigidity, translucency, surface texture, edge sharpness, and color, alongside a hierarchical decision framework instructing the model to assess fragment thickness first, then surface curvature, then optical properties. This level of prompt specificity was necessary to prevent the model from defaulting to generic plastic descriptions, but it also revealed a fundamental limitation: despite the detailed guidance, model outputs remained heavily anchored to whichever surface-level visual feature dominated the image, and the embedded reasoning framework did not reliably translate into correct classification. The prompt sensitivity of all three models constitutes an important reproducibility concern for any field deployment scenario, where prompt versions would need to be versioned and validated as carefully as any other methodological parameter.

### 4.2. Summary: Why Generative Models Fell Short

Taken together, the three generative models confirmed that zero-shot instruction-based polymer classification from RGB field imagery is not a solved problem at current model scales and prompt designs. All three models shared a common failure structure: classification decisions were driven by coarse visual heuristics, translucency, surface regularity, and perceived thickness, which do not reliably discriminate between polymer classes. The prompt-dependence of all three models, combined with their poor confidence calibration and inability to generalize beyond dominant training-set visual patterns, established a clear need for an alternative approach that does not rely on language generation as the classification mechanism. Table 2, summarizes the key failure modes of generative models.

### 4.3. Performance and Remaining Limitations

CLIP substantially outperformed all three generative models on this dataset, demonstrating that visual similarity matching in a rich pretrained embedding space is a more effective approach than zero-shot language reasoning for this classification task. The improvement is attributable to CLIP’s ability to match images on the basis of learned visual feature geometry rather than surface-level heuristics expressed through language, a distinction that matters precisely because the diagnostic features that separate polymer classes in this dataset are subtle, spatially distributed texture and reflectance properties rather than the gross morphological features that generative models tend to anchor on.

However, CLIP’s performance advantage comes with an important qualification that limits its direct applicability to real-world monitoring. The prototype-based classification approach is inherently tied to the visual distribution of the ten reference images used to construct each class prototype. In this evaluation, reference and test images were drawn from the same dataset, captured under the same field conditions, with the same camera, at the same site, across the same three-month collection period. CLIP’s prototype-based approach involves no parameter optimization over the dataset. Class prototypes are static embedding averages and model weights remain fixed throughout, ruling out overfitting in a classical sense. However, shared imaging conditions may passively reduce the domain gap between reference and test embeddings, and we acknowledge that this could contribute to the observed result. The 93.1% figure should therefore be interpreted as a within-distribution estimate of classification accuracy rather than a measure of generalizability across imaging environments. In a genuine deployment scenario, where a monitoring system trained on Tunisian coastal samples is applied to images from a different Mediterranean site, captured with a different device, in different seasons, or under different weathering regimes, the prototypes may no longer adequately represent the visual appearance of the target polymer classes, and classification accuracy would be expected to degrade. This domain gap between reference and deployment conditions is the central remaining challenge for CLIP-based microplastic monitoring and represents the primary direction for future methodological development. The embedding overlap observed between visually similar polymer classes, particularly PET and LDPE, can be attributed to shared surface-level optical properties that CLIP’s visual encoder cannot fully disambiguate from RGB imagery alone. Both polymers can appear as thin, semi-translucent fragments with smooth surfaces and relatively clean edges. Since CLIP’s prototype embeddings are constructed entirely from RGB pixel data, fragments whose reflectance, translucency, and surface texture fall within overlapping regions of the embedding space will produce ambiguous cosine similarity scores across class prototypes. This is consistent with the broader constraint established in Section 3.2, that all models in this study were evaluated on visual appearance alone, without spectroscopic or chemical features that would provide polymer-specific discrimination independent of surface condition. Furthermore, additional validation will be required to determine how image-based polymer classification can be integrated with ecological, toxicological, and exposure studies to support broader environmental and One Health applications.

An additional limitation concerns the interpretation of polymer classification results within broader environmental and public health contexts. While accurate identification of microplastic types may contribute to environmental monitoring efforts, the present study does not evaluate polymer toxicity, contaminant transport, biological uptake, trophic transfer, ecological effects, or human exposure pathways. Consequently, the performance metrics reported here should be interpreted as measures of image-based classification capacity rather than indicators of ecological or public health risk.

## 5. Conclusions

### 5.1. Summary

This study set out to evaluate whether multimodal foundation models could automate the segmentation and polymer classification of marine microplastic debris collected under ecologically realistic field conditions along the Mediterranean coast of Sousse, Tunisia. The central finding is that performance depends critically on the type of model and the mechanism by which it performs classification, and that the gap between generative language-based reasoning and visual similarity matching is substantial for this specific task.

The three generative vision-language models evaluated: LLaVA-OneVision (7 B), Qwen2-VL (2 B), and Llama-3.2-Vision-Instruct (11 B), each demonstrated that zero-shot polymer classification from RGB field imagery cannot be reliably achieved through natural language reasoning at current model scales. Classification decisions were driven by coarse surface-level heuristics such as translucency and perceived thickness that do not reliably distinguish between polymer classes once environmental weathering has degraded the diagnostic visual signatures present under controlled conditions. A shared and operationally significant failure across all three models was confidence miscalibration. CLIP ViT-B/32 demonstrated a fundamentally different and substantially more effective approach, achieving 93.1% overall classification accuracy. By bypassing language generation entirely, CLIP eliminated the prompt sensitivity that limited the generative models and exploited a richer, more abstract representation of visual material properties.

These findings support the potential role of AI-assisted microplastic monitoring within a One Health framework. This perspective is consistent with emerging One Health literature that recognizes environmental surveillance as a foundational component for understanding potential links between ecosystem contamination, wildlife exposure, and human health outcomes [20,21]. The polymer-resolved classification capability demonstrated by CLIP, which enabled the processing of hundreds of images without spectroscopic instrumentation, manual sorting, or expert annotation at inference time, highlights the potential of vision-language models as scalable screening tools for environmental monitoring. Such approaches may facilitate more frequent and spatially distributed assessments of coastal microplastic contamination than traditional manual methods alone.

While this study did not evaluate polymer toxicity, biological uptake, trophic transfer, or human health outcomes, the ability to characterize polymer-specific contamination patterns may provide useful baseline information for future ecological and public health investigations. Consequently, AI-based classification should be viewed as a complementary monitoring approach that can support environmental surveillance and guide subsequent risk assessment efforts rather than as a direct measure of ecological or human health risk.

### 5.2. Limitations

Several limitations should be considered when interpreting the findings of this study and assessing the generalizability of its conclusions.

- Geographic specificity. The polymer composition, fragment size distribution, weathering state, and sediment matrix of microplastic accumulations vary substantially across Mediterranean coastlines as a function of proximity to urban areas, river inputs, maritime traffic, and prevailing current systems. Model performance observed here, particularly CLIP’s strong results, may not transfer directly to sites with different contamination profiles or polymer mixtures, and the prototype embeddings constructed from Tunisian coastal samples may not adequately represent the visual appearance of the same polymer classes at geographically distinct locations.
- Limits of prompt engineering. The extensive prompt engineering required to elicit even partially structured outputs from these models represents a reproducibility concern that would need to be addressed before any field deployment could be standardized.
- Dataset size and class balance. While the full annotated dataset of 1080 images represents a meaningful contribution to the field, the evaluation subset of 60 images (10 per class) is small relative to the complexity of the classification task. A larger and more balanced evaluation set would improve confidence in class-specific conclusions and reduce sensitivity to individual image characteristics.
- Zero-shot evaluation of generative models. The generative models were evaluated without task-specific fine-tuning, which likely represents a significant performance floor rather than a ceiling. Fine-tuned variants trained on domain-specific examples from the Tunisian coastal dataset would be expected to outperform the zero-shot baselines substantially, and the current results should not be interpreted as indicating that generative models are categorically unsuitable for this application.
- CLIP domain gap. CLIP’s strong performance was achieved on a test set drawn from the same dataset and imaging conditions as its reference prototypes. This within-distribution evaluation does not constitute validation of cross-site or cross-device generalization, and performance should be expected to degrade when the model is applied to images collected under different environmental or technical conditions.

### 5.3. Future Research

The results and limitations of this study point toward several concrete directions for future research that would advance the field toward operationally deployable, ecologically validated microplastic monitoring systems.

- Ecological risk integration and One Health monitoring frameworks. Future research integrating environmental monitoring, toxicological assessment, and exposure analysis will be necessary to determine whether AI assisted microplastic classification can support quantitative risk assessment within a One Health framework. Beyond classification accuracy, additional work is needed to develop the policy and management interface between AI based polymer monitoring and coastal health decision making. This may include the development of polymer resolved risk indices that incorporate polymer specific toxicological characteristics, contamination levels, proximity to fisheries and aquaculture zones, and empirically measured trophic transfer pathways in marine food webs. Integration of such risk indicators into national and regional One Health monitoring systems, including existing Mediterranean environmental reporting frameworks, could help translate environmental surveillance data into decision support tools for coastal management. However, these applications remain a future research direction and were not directly evaluated in the present study.
- Integration of spectroscopic data. While this study demonstrated the feasibility of RGB-only polymer classification, future work should systematically evaluate the performance gain achievable by integrating complementary modalities, particularly near-infrared (NIR) hyperspectral imaging, which provides polymer-specific spectral signatures that persist after surface weathering has eroded optical discrimination. A multimodal pipeline combining CLIP-style visual embeddings with NIR spectral features in a shared representation space could substantially reduce confusion between polymer pairs such as PET and LDPE, PP and HDPE, which share broad RGB appearance but diverge sharply in their NIR absorption profiles.
- Cross-regional validation. The most immediate priority is to test whether models trained or prototyped on Tunisian coastal samples generalize to other Mediterranean sites where polymer composition, fragment weathering state, and sediment backgrounds differ systematically. A cross-regional validation study using a standardized imaging protocol and shared annotation schema would establish whether CLIP-based monitoring can support harmonized, basin-wide pollution assessments or whether site-specific prototype recalibration is required for each deployment location.

## Figures and Tables

**Table 1:** Summary of Evaluated Models.

Model	Params	Paradigm	Quantization	Output Schema
LLaVA-OneVision (Qwen2-7B)	7 B	Generative, zero-shot	Float16	has_plastic, predicted_label, confidence, reason
Qwen2-VL-2B-Instruct	2 B	Generative, zero-shot	4-bit NF4	type, polymer, confidence, reasoning
Llama-3.2-11B-Vision-Instruct	11 B	Generative, zero-shot	4-bit NF4	has_plastic, predicted_label, confidence, reason
CLIP ViT-B/32	86 M	Prototype cosine similarity	None	Per-class cosine similarity scores

**Table 2:** Comparison of generative model key failure modes.

Model	Prompt Style	Key Failure Mode
LLaVA-OneVision	Minimal structured JSON	Systematic PET/PP confusion; confidence always 1.0
Qwen2-VL	Detailed visual decision guide	Collapsed onto HDPE; macro F1 = 0.07
Llama-3.2-Vision	Structured JSON + UNKNOWN option	Every plastic predicted category collapsed onto HDPE

## Data Availability

The data of this article will be made available by the authors on request.

## Supplementary Materials

The following supporting information can be downloaded at: https://www.mdpi.com/article/10.3390/ijerph23070929/s1, Table S1: Qwen2-VL model performance metrics; Table S2: CLIP model: Classification Report; Figure S1: Llama-3.2-Vision-Instruct model: Prediction label confusion matrix; Figure S2: CLIP model: Prediction label accuracy; Figure S3: CLIP model: Heatmap of the misclassified labels. The following supporting information can be found at: https://fromtrash2treasure.metabolismofcities-llab.org/1254-2/ (accessed on 3 July 2026).
