# Supplementary material for: Can AI Reliably Identify Marine Microplastics in Wildlife? Assessing Multi-Modal Foundation Models for Polymer Classification with Minimal Training
Source: Int J Environ Res Public Health. 2026 Jul 20;23(7):929. doi: 10.3390/ijerph23070929 (PMC13410724; doi:10.3390/ijerph23070929)
Supplement: Supplementary file 1 [file ijerph-23-00929-s001.zip › ijerph-4318079-supplementary.pdf]

Table S1. Qwen2-VL model performance metrics.

Class	Precision	Recall	F1_score	Support
PS	0	0	0	10
PA	0	0	0	0
HDPE	0.2857142857	1	0.4444444444	8
PET	0	0	0	0
LDPE	0	0	0	10
PP	0	0	0	0

Table S2. CLIP model: Classification Report.

Class	Precision	Recall	F1-score	Support
HDPE	0.905982906	1	0.9506726457	106
LDPE	0.735042735	0.9772727273	0.8390243902	88
PA	1	1	1	122
PET	0.9782608696	0.8333333333	0.9	108
PP	1	0.8	0.8888888889	110
PS	1	0.9803921569	0.9900990099	102
accuracy	0.9308176101	0.9308176101	0.9308176101	0.9308176101
macro avg	0.9365477518	0.9318330362	0.9281141558	636
weighted avg	0.9439781802	0.9308176101	0.9317190622	636

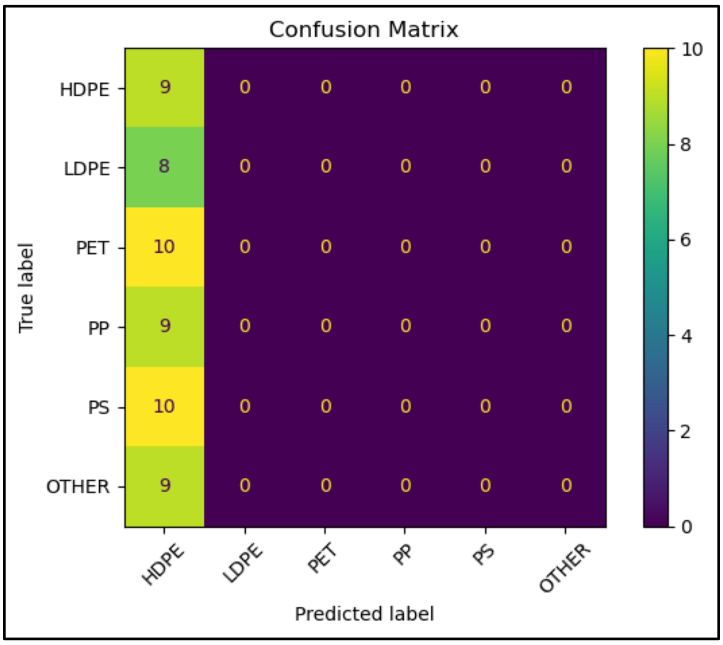

Figure S1. Llama-3.2-Vision-Instruct model: Prediction label confusion matrix.

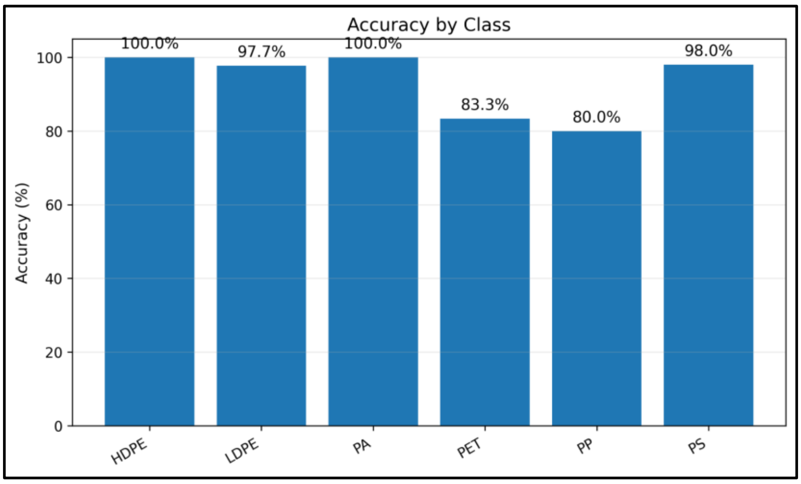

Figure S2. CLIP model: Prediction label accuracy.

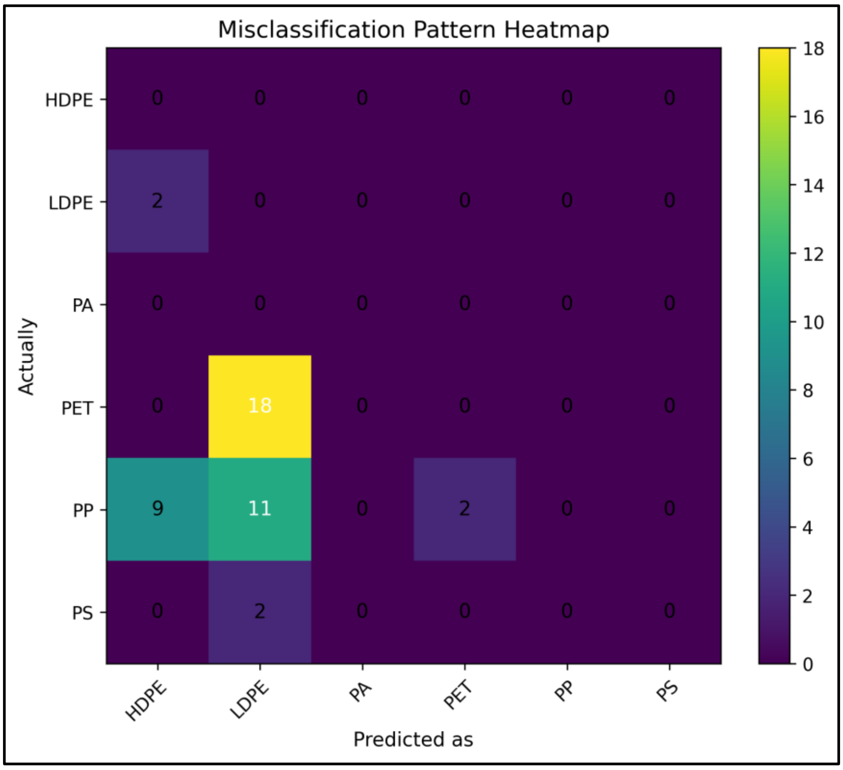

Figure S3. CLIP model: Heatmap of the misclassified labels.
